# Embedding MRI information into MRSI data source extraction improves brain tumour delineation in animal models

**DOI:** 10.1371/journal.pone.0220809

**Published:** 2019-08-15

**Authors:** Sandra Ortega-Martorell, Ana Paula Candiota, Ryan Thomson, Patrick Riley, Margarida Julia-Sape, Ivan Olier

**Affiliations:** 1 Department of Applied Mathematics, Liverpool John Moores University, Liverpool, England, United Kingdom; 2 Centro de Investigación Biomédica en Red en Bioingeniería, Biomateriales y Nanomedicina (CIBER-BBN), Cerdanyola del Vallès, Spain; 3 Departament de Bioquímica i Biologia Molecular, Unitat de Biociències, Universitat Autònoma de Barcelona, Cerdanyola del Vallès, Spain; 4 Institut de Biotecnologia i de Biomedicina, Universitat Autònoma de Barcelona, Cerdanyola del Vallès, Spain; Istituto Italiano di Tecnologia, ITALY

## Abstract

Glioblastoma is the most frequent malignant intra-cranial tumour. Magnetic resonance imaging is the modality of choice in diagnosis, aggressiveness assessment, and follow-up. However, there are examples where it lacks diagnostic accuracy. Magnetic resonance spectroscopy enables the identification of molecules present in the tissue, providing a precise metabolomic signature. Previous research shows that combining imaging and spectroscopy information results in more accurate outcomes and superior diagnostic value. This study proposes a method to combine them, which builds upon a previous methodology whose main objective is to guide the extraction of sources. To this aim, prior knowledge about class-specific information is integrated into the methodology by setting the metric of a latent variable space where Non-negative Matrix Factorisation is performed. The former methodology, which only used spectroscopy and involved combining spectra from different subjects, was adapted to use selected areas of interest that arise from segmenting the T2-weighted image. Results showed that embedding imaging information into the source extraction (the proposed semi-supervised analysis) improved the quality of the tumour delineation, as compared to those obtained without this information (unsupervised analysis). Both approaches were applied to pre-clinical data, involving thirteen brain tumour-bearing mice, and tested against histopathological data. On results of twenty-eight images, the proposed Semi-Supervised Source Extraction (SSSE) method greatly outperformed the unsupervised one, as well as an alternative semi-supervised approach from the literature, with differences being statistically significant. SSSE has proven successful in the delineation of the tumour, while bringing benefits such as 1) not constricting the metabolomic-based prediction to the image-segmented area, 2) ability to deal with signal-to-noise issues, 3) opportunity to answer specific questions by allowing researchers/radiologists define areas of interest that guide the source extraction, 4) creation of an intra-subject model and avoiding contamination from inter-subject overlaps, and 5) extraction of meaningful, good-quality sources that adds interpretability, conferring validation and better understanding of each case.

## Introduction

**Fig 1:**
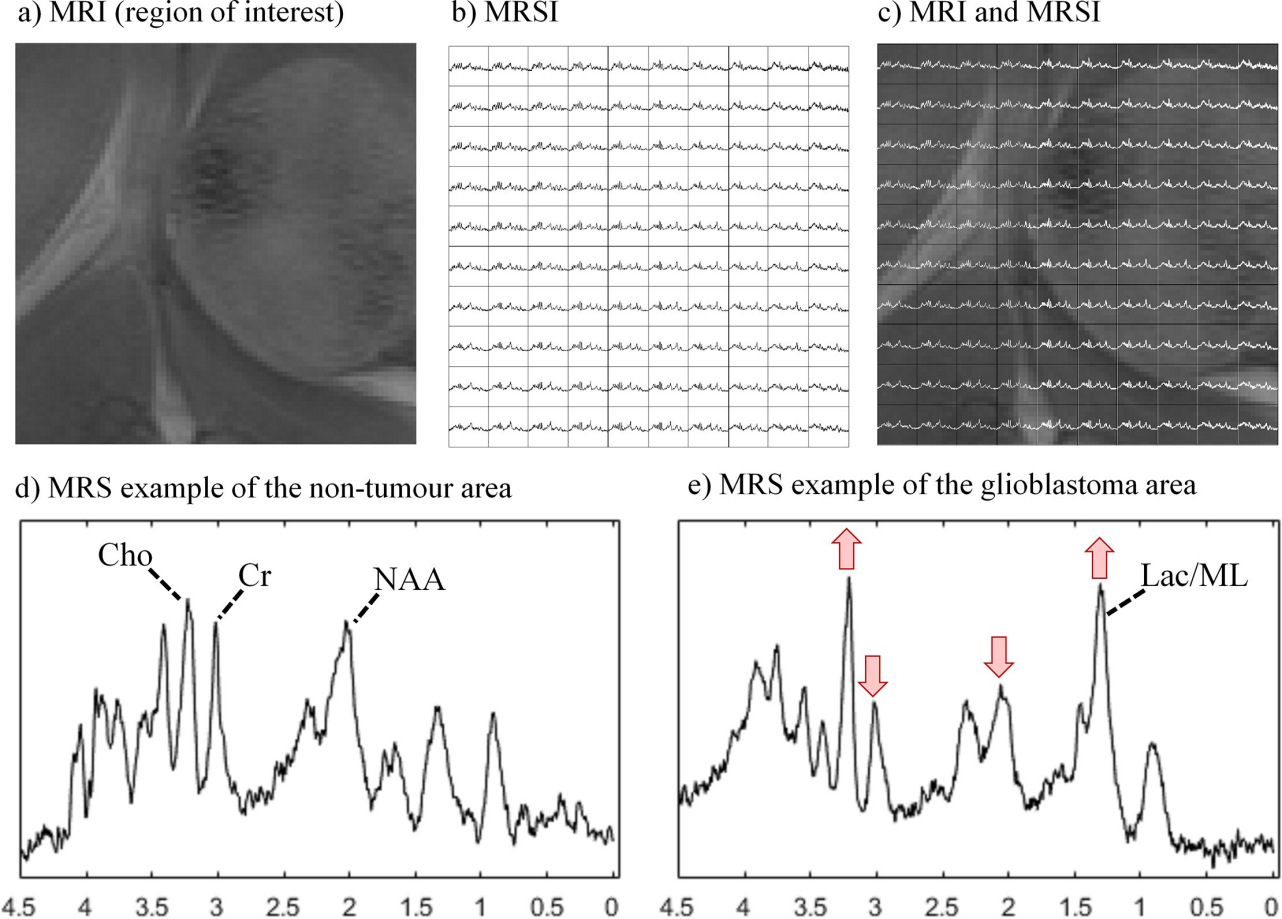
Two magnetic resonance approaches, MRI and MRSI, acquired in a murine glioblastoma model. a) Region of interest of the MRI. b) 10-by-10 MRSI grid of voxels, showing the metabolic composition of the tissue at voxel level. c) MRSI grid superimposed on the MRI image. d) MRS example of the non-tumour area of this mouse, indicating the position of a number of relevant metabolites: choline (Cho), creatine (Cr) and N-acetyl aspartate (NAA). e) MRS example of the non-tumour area of this mouse, including lactate/mobile lipids (Lac/ML), and highlighting the changes that occur to these relevant metabolites.

Magnetic Resonance (MR) is widely used for non-invasive investigations of brain tumours, in particular *in vivo* diagnosis and grading, surgical planning and assessment of response to therapy. It is generally applied as MR imaging (MRI), see Fig 1(A), which provides a morphologic characterisation of tissues, and it is the modality of choice in diagnosis, aggressiveness assessment and follow-up. However, there are examples (i.e. malignant gliomas) where current imaging techniques lack diagnostic accuracy [1]. In contrast, MR spectroscopy (MRS) provides biochemical information, resulting in a precise metabolomic signature of the target tissue, which enables the identification of a wide array of molecules present in tissues. MR spectroscopic imaging (MRSI), see Fig 1B and 1C, produces a spatial distribution of these metabolomic profiles, and thus delivers information about the spatial localisation of molecules [2,3]. Typically, an MRSI acquisition of the brain consists of a spectral grid of varying dimensions (e.g. 10-by-10, 12-by-12) superimposed on the image, covering only a part of the full image.

Glioblastoma (GB) is the most frequent malignant intra-cranial tumour, having a very poor prognosis. Currently there is no cure for the disease. The standard treatment is surgery, with the aim of maximal safe tumour removal, followed by radio- and chemotherapy [4]. Despite this, due to the infiltrative nature of GB it is not always possible to assess the true degree of infiltration for a total tumour resection using conventional MRI [5]. Additionally, chemo- and radiotherapy only provide a small increase in the overall survival [6]. Eventually, most patients end up relapsing, and when this happens, they may be offered the possibility of starting with either second-line or experimental treatment approaches. One of the problems at this stage is the early assessment of progression using non-invasive methods such as MRI, which may be inaccurate for some patients (e.g. distinguishing true progression from pseudo-progression [7]). In this sense, non-invasive tools able to accurately distinguish the compromised area, which in turn would allow early prediction of relapse, would be of maximal benefit.

Pattern recognition and machine learning methods have been extensively applied to MR data of brain tumours, to assist with different clinical issues, from diagnosis and prognosis of several pathologies, to delineation of tumour masses [8–11]. Most studies have been performed on single-voxel MRS [12–16], where a single spectrum is acquired from the pathological area. Even though substantial advances have been achieved, there are still challenges from the methodological point of view that need to be addressed. One of them is the ability to combine, in a principled manner, information from two different MR approaches such as images and spectra. As can be seen in Fig 1, the resolution of the MRSI (i.e. number of voxels of each acquisition) is considerably lower than the resolution of the MRI, which is at the pixel level. This combination of MR images and spectra poses an important challenge, as it is necessary to deal with different types of signals that also have different resolutions [17,18].

In biomedical research, animal (preclinical) models are useful for testing new drugs or treatments. One of the main advantages is the possibility of performing post-mortem studies–for example in the case of brain studies, to excise the whole brain and perform a detailed histopathological analysis. This allows the validation of imaging techniques and mathematical analyses or transformations of those, in contrast to patient studies, in which such validation approaches are not feasible due to obvious ethical restrictions.

The clinical value of a multivariate statistical analysis based on multiparametric MRI and MRSI for the non-invasive analysis of brain tumours has been previously assessed in a number of publications, such as [9,10,19]. They show that results combining MR approaches are more accurate and have superior diagnostic value compared with single approach. Most of those works used supervised mathematical models that explicitly require class information, i.e. tumour type and grade. This is often achieved in an *ad-hoc* manner by concatenating or mixing selected characteristics from each approach (that can be MR images and spectra), such as in [3,20,21]. A more recent study [22] makes use of Structured Data Fusion (SDF) [23], which could be potentially used for a more appropriate coupling of the information by joining the factors that are obtained in the factorisation process. However, authors in [22] do not seem to have used SDF in that way, but instead chose to concatenate the information as in the aforementioned studies. Providing multiparametric MRI and MRSI approaches in a principled manner may help to overcome the instability in the tissue segmentation that may arise from intrinsic mixing in data space.

Therefore, the purpose of our study was to develop a new methodology for embedding morphological information from MR images into the MRSI analysis of brain tumours in animal models, using a Semi-Supervised approach to Source Extraction (SSSE). We applied this approach to retrospective MR data from an orthotopic murine GB model (GL261 GB growing into C57/BL6 mice), widely used in preclinical research, which mimics most of the human GB features [24]. In order to validate the MRI+MRSI fusion, preclinical data allowed us to assess the quality of the tumour segmentation in comparison to a third, independent technique (histopathology). We applied our methodology to both control and treated tumour-bearing mice.

## Materials and methods

### Ethics statement

No ethics approval was required for the current retrospective study. All studies with mice were approved previously by the local ethics committee [*Comissió d’Ètica en l’Experimentació Animal i Humana* (CEEAH). Available: http://www.uab.cat/etica-recerca/. Last accessed 29/06/2018], according to the regional and state legislation (protocol DARP-3255/CEEAH-530). Mice were periodically subjected to welfare inspections to check for any early symptoms of suffering and an objective scale for signs and symptoms was established. Mice were obtained from Charles River Laboratories (France) and housed at the animal facility of the *Universitat Autònoma de Barcelona* (*Servei d’Estabulari*).

### MR studies

MR studies were carried out at the joint nuclear MR facility of UAB and CIBER-BBN, Unit 25 of NANBIOSIS (www.nanbiosis.es), with a 7 Tesla horizontal magnet (BioSpec 70/30, Bruker BioSpin, Ettlingen, Germany). Details of the acquisition parameters can be found in the Supporting Information file.

MRSI data were processed as described in [25,26]. The MRSI data grid was formed by an array of 10×10 voxels (MR spectrum from each voxel contained 692 data points), with an in-plane resolution of 0.55×0.55 mm and a 1 mm slice thickness in the 3rd dimension [25]. This volume of interest was manually positioned approximately in the centre of the brain, based on the reference image, in a way that it would include most of the tumour mass and also part of the normal/peritumoural brain parenchyma.

The study was performed on retrospective data already acquired. Only the spectral information (MRSI) from these data had been used in previous pattern recognition studies [25–28], performed for other purposes. Reference T2w MRI for all mice had not been used previously in any of the analyses, except for providing the anatomic overlay for the MRSI data. This is the first time that the images (MRI) were also part of the pattern recognition analysis for these mice.

### Datasets

#### Pre-treatment data for analysis of tumour *vs*. non-tumour (groups A and B)

In this section, we describe the control (untreated) mice data that was used in this study to evaluate the ability of SSSE to delineate the tumour mass. This data is summarised in Table 1 and includes:

A MRI and MRSI (short TE, 12ms) data of six mice from [25]. The MRSI data in the mentioned study had been used for the purpose of discriminating the tumour from the healthy tissue, in a fully unsupervised way.
B MRI and MRSI (short TE, 14 ms) data of five mice from [26,28]. The MRSI data in the mentioned studies had been used as control (untreated) group to assess response to therapy, in a semi-supervised way.

**Table 1:** Data used for analysis in this study.

Group	Analysis in previous studies	References	Unique ID of mice (D: day post-inoculation)	Number of mice
A	Performed on untreated cases	[25,27]	C69 (D: 15), C71 (D: 16), C32 (D: 16), C179 (D: 17), C233 (D: 17), C234 (D: 17)	6
B	Performed on untreated cases	[26,28,29]	C255 (D: 14), C288 (D: 18), C520 (D: 18), C529 (D: 18), C583 (D: 18)	5
C	Performed on treated cases, longitudinal study	[26]	C819 (D: 10, 15, 18, 21, 25, 30, 33, 41, 45), C821 (D: 10, 15, 18, 21, 25, 30, 33)	2

This table includes the references to original studies with this data, unique ID of animals, and their number of individuals.

#### Follow-up, longitudinal data for analysis of tumour *vs*. non-tumour (group C)

In this section, we describe the follow-up, longitudinal data that was used in this study. This is data obtained from mice under temozolomide (TMZ) treatment with the administration schedule described in [28] and in the Supporting Information file.

The aim was to assess SSSE’s ability to produce an accurate delineation of the tumour mass, as well as recognise the volume changes at different time points, and tumour response to therapy. This data is also summarised in Table 1 and includes:

C MRI and MRSI (short TE, 14 ms) data of two treated mice from [26]. Again, only the MRSI data had been used previously. For this study, we selected the two mice receiving treatment for the longest period from [26] (survival time of 45 and 34 days, and with 9 and 7 MR explorations, respectively).

### Histopathology

After the MRSI study (or at after MRSI endpoint in the Group C cases), the animals were euthanised according to the ethics protocol by experienced personnel and the brains were collected and analysed by histopathology as described in [28]. Caspase 3 immunohistochemical staining was used for detecting apoptosis. Ki67 immunohistochemical staining was used to determine the spatial proliferating population of cells in each tumour mass [25,30], calculated as a proliferation index (PI). In this particular murine model, and according to the veterinary pathologist, PI > 30% would correspond to a safe threshold for identifying the solid tumour region, whereas a PI ≤ 5% would correspond to definitely non-tumour (excluding reactive gliosis and other phenomena). This is in agreement with other studies with murine glioma, in which a PI of 23.9% was found in tumour core, and 9.6% in tumour periphery [31].

The evaluation of necrosis was performed by the histopathologist on the haematoxylin and eosin stained slides. Different features were considered: isolated necrotic cells, moderate amounts of eosinophilic debris and large empty spaces. For each tumour, the percentage of the tissue section affected was semi-quantified. When a tumour showed less than 20% of the mentioned necrotic features, a low grade of necrosis was assigned, while high grade of necrosis was assigned to those having more than 20% of the features. More details are included in [26].

**Fig 2:**
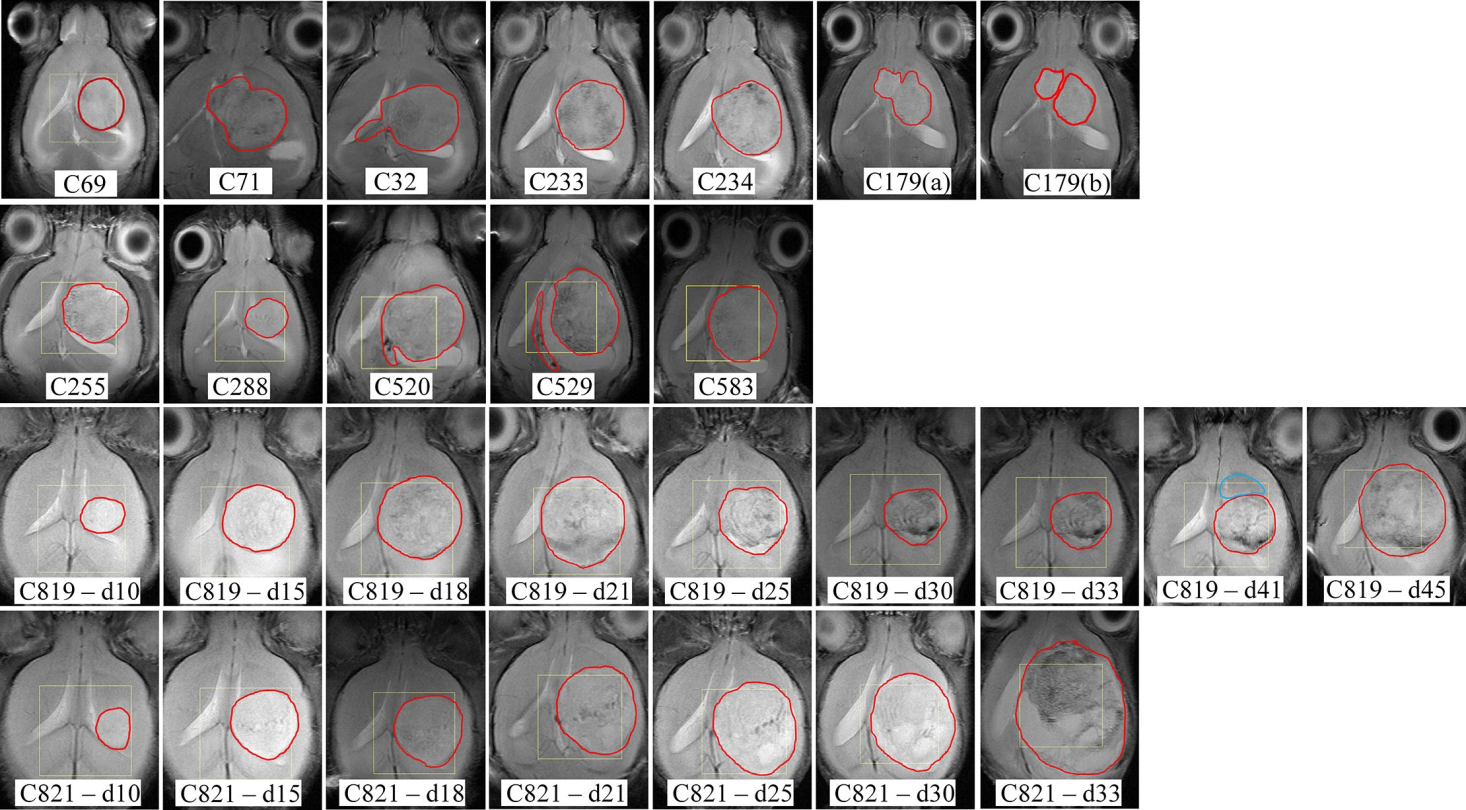
Delineation of the tumour area used as gold standard in this study. Images were produced *a posteriori* by the preclinical bioimaging expert for Groups A (first row), B (second row), and C (third and fourth rows). Two versions, (a) and (b), are available for C179, to study the main tumour mass first, and the two masses later, considering they have different proliferation indices, i.e. the rostral mass (“secondary mass”) had a 5% < PI ≤ 30 while the caudal mass (“core mass”) had a PI > 30%. Additionally, for C819 at day 41, a second (blue) area was highlighted as abnormal.

Using all the available histopathological information such as Ki67 (which is information obtained *a posteriori*, or *ex-vivo*), the preclinical bioimaging expert produced a set of images with the aim of using them as the gold standard for this study. These images are compiled in Fig 2.

### Non-negative Matrix Factorisation for source extraction

In Non-negative Matrix Factorisation (NMF) methods [32,33] the non-negative data matrix X is approximately factorised into two non-negative matrices: the matrix of sources or data basis S and the mixing matrix H. The product of these two matrices provides an approximation to the original data matrix in the form X≈SH. There are different NMF variants, which mainly arise from using different cost functions for computing the divergence between X and SH. While NMF describes the observed data with positive-only mixtures of the latent variables or data sources, this does not apply to long echo times where spectral phase-related signal modulation frequently results in negative values in the lactate and alanine regions [12].

Convex Non-negative Matrix Factorisation (Convex-NMF) [34] is a variant of NMF that imposes a restriction over the source matrix S to be a convex combination of the input data vectors. This restriction significantly improves the quality of data representation of S. Unlike standard NMF, Convex-NMF applies to both nonnegative and mixed-signed data matrices. What this means in practice is that: a) the data are described by positive-only mixtures of the mixed-signed sources; and b) the sources, or latent variables, are also positive-only mixtures of the data. This makes it easier to interpret both the mixing and unmixing processes.

### Semi-supervised methodology for source extraction in MRSI data

The semi-supervised methodology proposed in [13] involves three main stages and, in a nutshell, can be described as follows:

- Definition of a Fisher Information (FI) metric to model pairwise similarities and dissimilarities between data points, using a Multi-Layer Perceptron (MLP) classifier to estimate the conditional probabilities of class membership.
- Approximation of the empirical data distribution in a Euclidean projective space in which we can apply NMF-based techniques. Multi-dimensional Scaling (MDS) is one of the algorithms proposed to do this mapping while retaining the distance structure generated by the FI matrix.
- Application of Convex-NMF for the source decomposition of the data, which includes the identification of the underlying sources and the calculation of the corresponding mixing matrix.

This semi-supervised methodology was previously applied to *single-voxel* data in [13], and *multi-voxel* data in [26], although in a slightly different way. Both studies involved using the labels provided by experts and combining spectra from different subjects to create a training dataset in which the sources were extracted.

### Proposed methodology to embed MRI information into the source extraction

**Fig 3:**
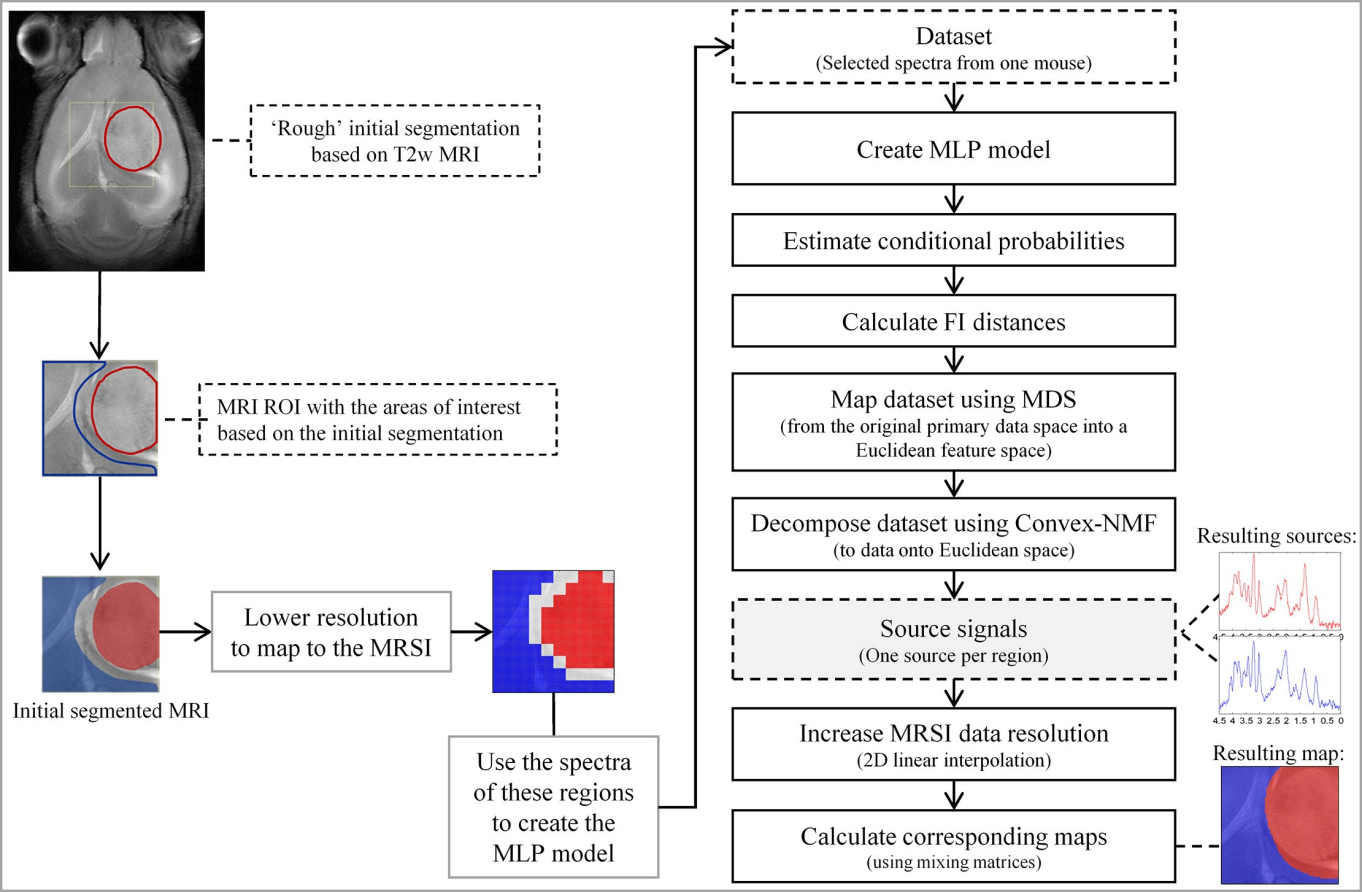
Diagram of SSSE. Details of the methodology proposed in this study for an extraction of sources in a semi-supervised way, informed by knowledge hauled out from the T2w MRI.

The proposed methodology, SSSE, builds upon the semi-supervised method proposed in [13] for the extraction of relevant sources, which guides the source extraction in the direction of provided class labels. In this new approach (see Fig 3), instead, we use the areas that arise from segmenting the MRI (i.e. T2w images), hence using the normal parenchyma/peritumoral/ventricle/tumour structures identified by MRI. We recommend this initial segmentation of the image to be performed manually by a researcher (e.g. radiologist, clinician, data analyst, etc.), but other automatic approaches can also be considered.

Importantly, the semi-supervised nature of the proposed methodology has the benefit that it allows using only partial regions, so that areas of uncertainty can be left outside the initial segmentation, while allowing for concentration on the areas of maximum interest.

Following the initial selection of the areas of interest (or segmentation), we compute the posterior probability of each class (or segmented region) for each pixel. As mentioned previously, our choice of estimator of these probabilities, p(c|x), is a Multi-Layer Perceptron (MLP), which is a feed-forward artificial neural network. This is a semi-parametric non-linear probabilistic model of class membership, for which a FI metric can be derived [35]. The parameters of the MLP were set as follows: We used one hidden layer of 6 nodes plus the output layer. The initial weights and bias were generated randomly in the interval [–1, 1], the learning rate was set to 0.05, the momentum constant to 0.9, and weight decay regularization was used to limit the size of the weights (lambda constant set to 0.01), which should encourage the creation of simpler models with better generalisation capabilities. We sought model convergence by allowing the MLP to iterate over a number of iterations (maximum epochs of 2000), until the error was smaller than 5e-3. The models were trained using 75% of the instances for training and the rest for test.

After calculating the posterior probability of each class for each pixel, as the resolution of the T2w image is higher than the one of the MRSI, we implement a voting system to bring the resolution of the T2w image down to the spectroscopic imaging. Then, we train the neural network model using the spectra from the MRSI and the labels provided by the segmentation, targeting the computed posterior probability of each class. From this point onwards, the rest of SSSE is similar to the semi-supervised methodology proposed in [13], but applied intra-subject (i.e. to an individual mouse) as opposed to a number of them (as in our previous study [13]). Final resulting maps with the produced segmentations were linearly interpolated to bring them to the resolution of the image, as in [25].

It is important to stress that SSSE was designed to create a model of an individual case, as the purpose was to better understand and reflect the characteristics of each single patient. Hence, in order to avoid contamination from inter-subject overlaps, SSSE does not involve combining spectra from different subjects, thus focusing on intra-subject variation. What this means in practice is that every single case, C_i_, was studied independently from the others (even excluding information from the same case acquired on a different day). From this data of case C_i_, a proportion (detailed previously) was used for the initial training step as required by the methodology, and from there the rest of the proposed pipeline is unsupervised.

### Evaluation of the results

To evaluate whether embedding information from the MRI into the data source extraction improves the quality of the tumour delineation, we compare SSSE segmentations with the ones obtained without prior knowledge (Convex-NMF). We also compared SSSE with the approach proposed by Sauwen et al. in [22], as they both bear some similarities in their aim to propose a semi-supervised / semi-automated method for the segmentation of brain tumours. For the latter comparison, some considerations had to be made to allow for a fair comparison of both methods (please see details in the Supporting Information file). The three approaches will be tested against the gold standard (see Fig 2, and the Histopathology section for more details).

As measures used for comparison, we start by calculating the sensitivity and specificity of detecting the tumour regions. The sensitivity, or true positive rate, is calculated as TP/(TP+FN), where true positive (TP) are tumour pixels correctly labelled as tumours; and false negative (FN) are the tumour pixels labelled as non-tumours. The specificity, or true negative rate, is calculated as TN/(TN+FP), where true negative (TN) are the non-tumour pixels correctly identified as non-tumours, and false positive (FP) are the non-tumour pixels labelled as tumours. A related measure frequently used in this area [36] for comparing the similarity of two samples is the Dice score coefficient (DSC), also known as the Sørensen–Dice coefficient [37], in order to show the effectiveness and robustness of proposed approach. This is calculated from the values of TP, FP and FN, as follows:

$$DSC=\frac{2TP}{2TP+FP+FN}$$ Eq 1

As an overlap-based metric such as DSC can be dependent on the segmentation size, we also calculate two distance-based metrics: the Euclidean distance, and the Hausdorff distance. These distances were calculated between each of the resulting images and the corresponding one from the preclinical bioimaging expert, considered the gold standard in this study. The purpose was to determine how far (based on these distances) from the gold standard was the estimation of the tumour area when using each of the benchmarked approaches (i.e. Convex-NMF, Sauwen’s and SSSE).

The Euclidean distance was calculated as in [38]. Hence, a pair of images E (estimated) and G (gold standard) having feature vectors f^E^ and f^G^, respectively, have the following distance:

$$Euc\_dist(E,G)=\sqrt{\sum_{i=1}^{n}{\left(f_{i}^{E}-f_{i}^{G}\right)}^{2}}$$ Eq 2

Where n is the number of voxels in the images. The distance between two identical images is zero, i.e. Euc_dist(G,G) = 0; and the larger the value (distance), the bigger the difference between them.

Turning the distance measure into a shape similarity score that is easier to interpret, we calculated then the number of pixels that match values in the two images with reference to the total number of pixels, giving us a percentage of success.

The Hausdorff distance, in turn, measures how far two sets of points are from each other and was used here to measure the most mismatched tumour delineation between the three segmentations (Convex-NMF, Sauwen’s and SSSE) with respect to the gold standard used. Let us consider B_E_ and B_G_ the boundaries of the tumour areas of the estimated and the gold standard images, respectively, of which we want to calculate the Hausdorff distance. It was then calculated as follows:

$$Haus\_dist(B_{E},B_{G})=\max\left\{\begin{array}{c} sup\\ e\in B_{E} \end{array}\;\begin{array}{c} inf\\ g\in B_{G} \end{array}dist(e,g),\begin{array}{c} sup\\ g\in B_{G} \end{array}\;\begin{array}{c} inf\\ e\in B_{E} \end{array}dist(e,g)\right\}$$ Eq 3

where *sup* represents the supremum and *inf* the infimum. This distance will be zero if and only if B_E_ and B_G_ have the same closure, i.e. both tumour areas are exactly the same.

Finally, the Kruskal-Wallis test was used to determine whether the results between the three approaches, i.e. Convex-NMF, Sauwen’s and SSSE, were statistically significant.

## Results

The presentation of results is divided into two sections. Firstly, we show the results of applying SSSE to the mice in Groups A and B, which include untreated and treated cases, respectively; and to the mice in Group C, belonging to a longitudinal study with treated mice. (Please refer to Table 1 for more details and references to these groups). This is followed by the evaluation of the presented results at the end of this section.

### Brain tumour delineation

**Fig 4:**
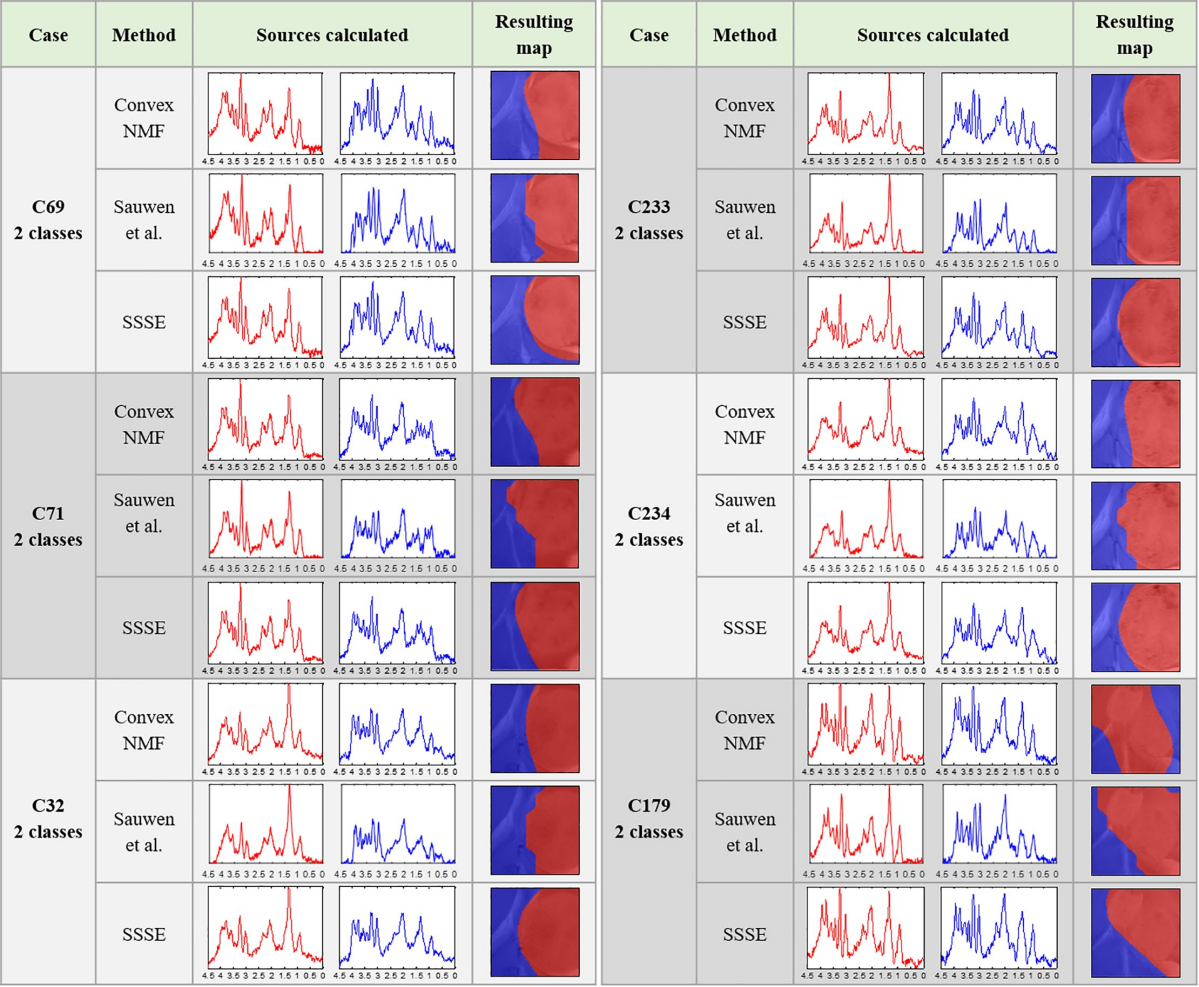
Results for the cases in Group A, for two classes.

Fig 4 shows that the sources obtained with the three approaches, for the different tissue types, are in some cases very similar to the naked eye, which is backed by correlations above 97% in most cases (e.g. comparing the red sources obtained with Convex-NMF, Sauwen’s approach and SSSE). However, even when subtle in some cases, these small differences between them are reflected in their corresponding colour-coded maps, indicating the tumour delineation.

**Fig 5:**
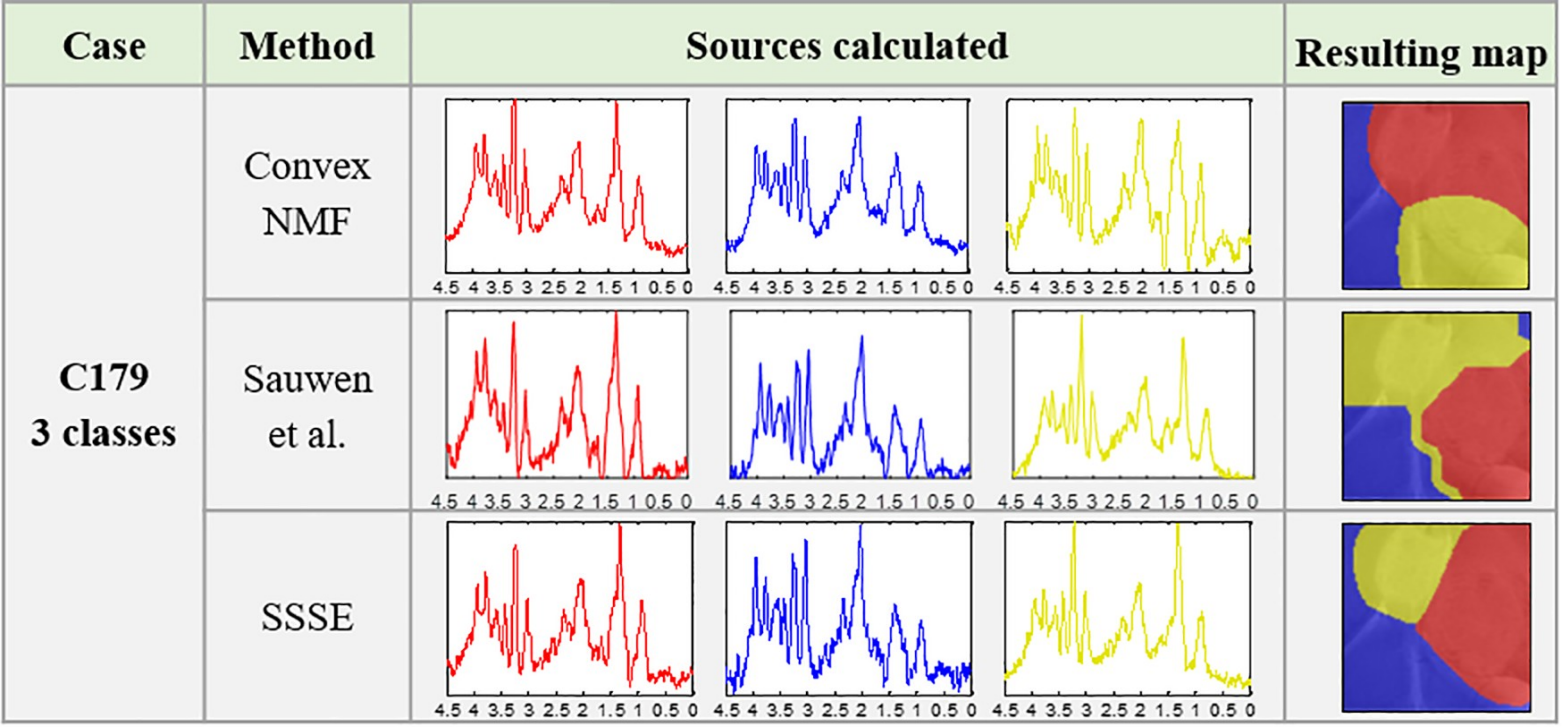
Results for case C179 for three classes, from Group A.

For mouse C179, we also extracted three sources, in order to check whether a higher number of sources could better represent its spatial heterogeneity–i.e. two tumour masses and three histologically different regions with PI ≤ 5% (normal brain), 5% < PI ≤ 30 (secondary mass) and PI > 30% (core mass)–see Fig 2. The results are presented in Fig 5. In this situation, both, the sources obtained and the resulting maps, were more visually different. For example, when comparing the red source (representing the main tumour mass) and the blue source (representing the non-tumour area) produced by SSSE with the equivalent sources produced by Convex-NMF, they showed high similarity between themselves (with a correlation of 97% for the main tumour mass and 95% for the normal tissue). However, the yellow source obtained was less similar (with a correlation of 93%). More importantly, these two sets of sources, i.e. red and yellow sources, were mainly representative of different areas of the brain, with Sauwen’s approach and SSSE providing a closer resemblance to the T2w image and the histopathological PI values of each mass (see Fig 2) than Convex-NMF. The latter uses mainly the red source to represent both tumour masses, according to the preclinical bioimaging expert, and the yellow source is covering both the solid tumour area and the non-tumour area.

**Fig 6:**
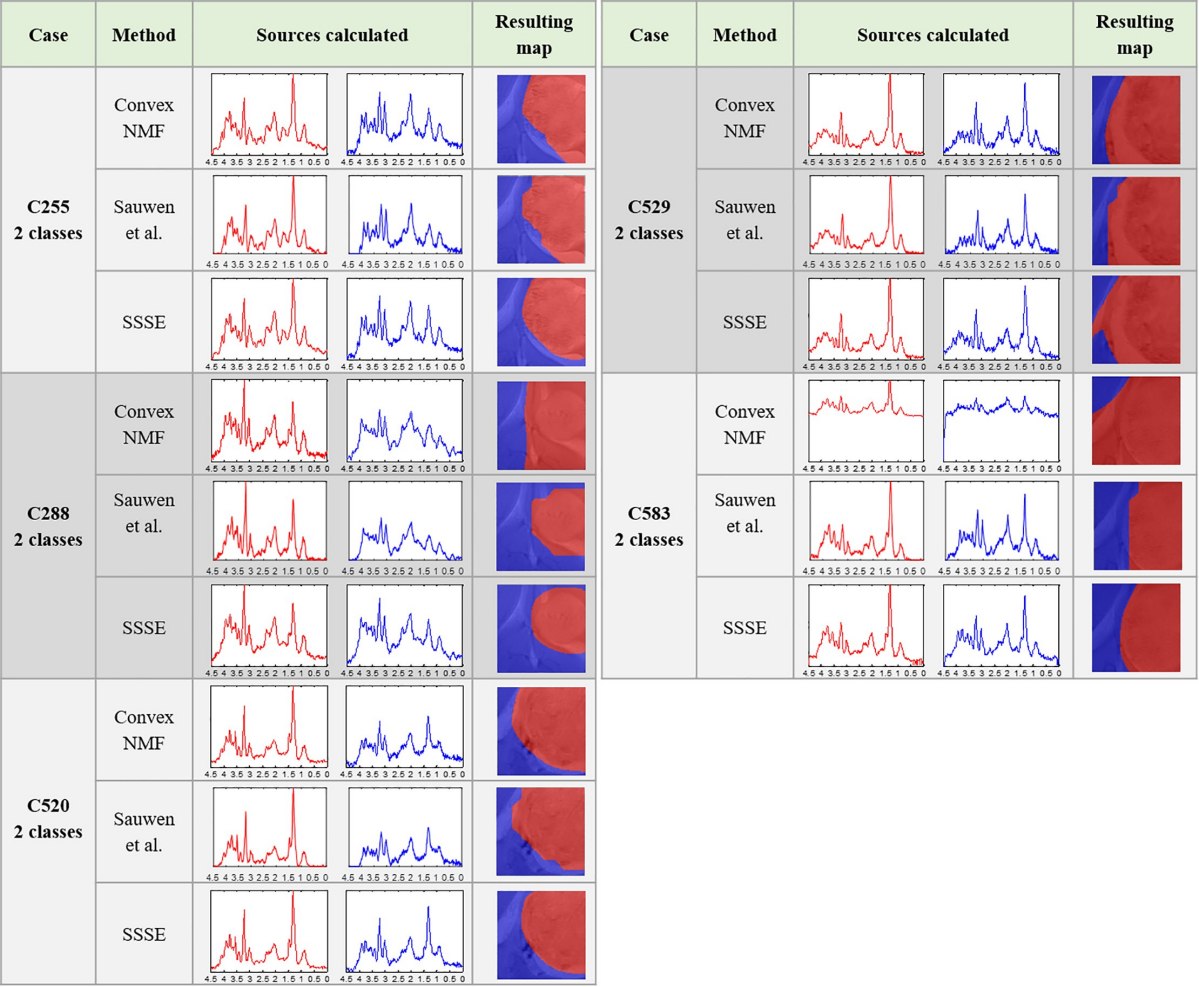
Results for the cases in Group B, for two classes.

Fig 6 shows the results obtained for the cases in Group B. These results were in line to those from Group A (excluding a few cases such as the three sources calculated for mouse C179), in the sense that the sources were not strikingly visually different, except for case C583, in which both the unsupervised tumour (red) and normal (blue) sources corresponded to patterns that matched with low signal-to-noise spectra. This is a problem that has been encountered and characterised before [39]. In the case of the non-tumour (blue) sources, they differed more than the tumour (red) source between the two approaches in all five mice.

**Fig 7:**
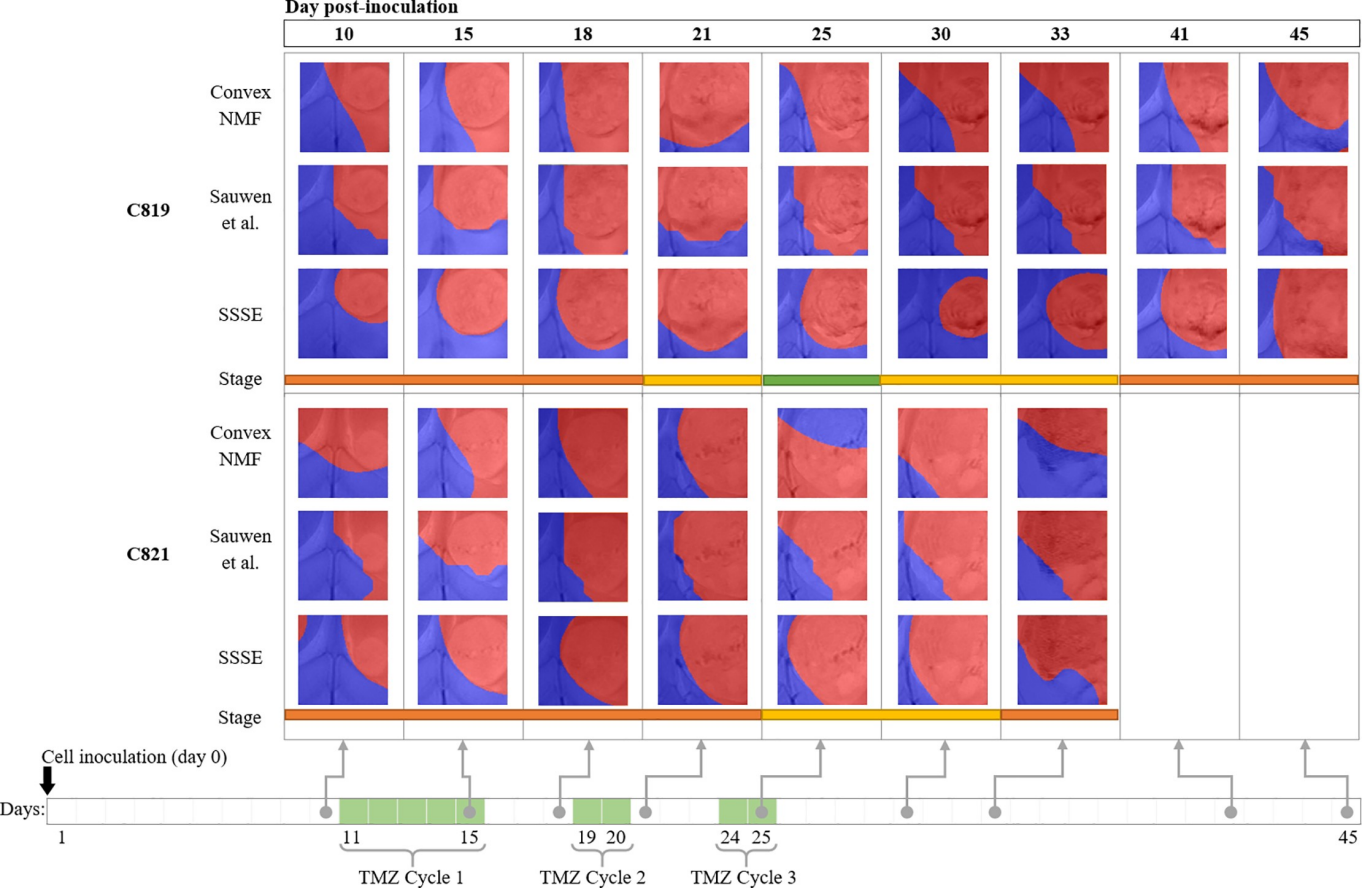
Results for the cases in Group C, longitudinal study. Under each case there is a colour-bar showing the response stage determined by the RECIST criteria throughout the therapy protocol (orange means progressive disease, yellow stable disease and green partial response). At the bottom it is indicated when the three TMZ cycles were administered.

Fig 7 compiles the resulting colour maps (tumour delineation) after applying Convex-NMF, Sauwen’s approach and SSSE to the two mice in Group C, at the different time points studied.

These colour-coded maps show how the volume of the tumour mass in each mouse changes as a result of their response to the three cycles of therapy. The RECIST criteria [40], which provides an indication of the response evaluation criteria in solid tumours, was determined for these two mice throughout the course of the treatment [26].

The adapted RECIST criteria were applied for mouse C819 [26] and in results from day 10 to day 18, the tumour was considered to be at the stage of progressive disease. At day 21, it entered the stable disease stage, remaining in a transient response state until day 25, when tumour shrinkage indicated partial response to the therapy. At days 30 and 33 the tumour was considered to be again in a stage of stable disease, gradually halting its response to the therapy and starting to regrow/relapse. Finally, at days 41 and 45 the tumour was considered again to be at the stage of progressive disease.

The adapted RECIST criteria for mouse C821 [26] indicated that the tumour was in the stage of progressive disease from day 10 to 21, in which the tumour grew from occupying ca. 15% of the area of the region of interest to nearly the 90% of it. This stage was followed by a short stable disease stage during days 30–35, followed again by another stage of progressive disease at the last day. A summarized explanation of the adapted RECIST criteria can be found in the Supporting Information file.

### Evaluation of the tumour delineation against the gold standard

The results presented in the previous section for the three approaches were quantitatively evaluated using two main criteria: i) their ability to delineate the tumour regions (therefore discriminating the tumour from the non-tumour regions) with high sensitivity and specificity; and ii) their ability to produce colour-coded maps that are as close as possible to the gold standard (see Fig 2), which in this study, it is the set of images provided by the preclinical bioimaging expert (see more details in Histopathology and Evaluation of Results sections from Methods).

Details of the sensitivity and specificity values corresponding to the ability of each approach to detect the tumour masses can be found in Tables A in S1 File (for Groups A and B), B in S1 File (for more details on mouse C179 from Group A), and C in S1 File (for Group C). These tables also include the Dice score coefficient calculated for the three methods against the gold standard. A summary of these results is presented in Table 2.

**Table 2:** Overall sensitivity/specificity and Dice score of the correct delimitation of the tumour mass.

Groups	Number of masses	Sensitivity / Specificity			Dice score		
		Convex-NMF	Sauwen et al.	SSSE	Convex-NMF	Sauwen et al.	SSSE
A and B	1	0.90 ± 0.08 / 0.68 ± 0.23	0.90 ± 0.08 / 0.76 ± 0.14	0.94 ± 0.05 / 0.82 ± 0.16	0.82 ± 0.11	0.84 ± 0.09	0.90 ± 0.05
A (C179)	2	0.70 ± 0.42 / 0.68 ± 0.04	0.87 ± 0.18 / 0.77 ± 0.11	1.00 ± 0.00 / 0.88 ± 0.05	0.45 ± 0.05	0.63 ± 0.13	0.82 ± 0.04
C	1	0.88 ± 0.17 / 0.60 ± 0.17	0.90 ± 0.08 / 0.72 ± 0.13	0.94 ± 0.10 / 0.81 ± 0.13	0.76 ± 0.13	0.82 ± 0.12	0.89 ± 0.10

Mean and standard deviation reported per group. First column indicates the group; second indicates the number of tumour masses; third to fifth columns include the sensitivity / specificity results for the three approaches, respectively; while sixth to eighth show their corresponding Dice scores. Shaded columns highlight the results obtained with SSSE.

Next, we present the results of the Euclidean distance between each of the three methods to the gold standard, followed by a shape similarity score (see Evaluation of the results in the Methods section), and the Hausdorff distance between the same set of images. The results for the individual cases in Groups A and B are shown in Table D in S1 File; while the results for Group C can be seen in Table E in S1 File. A summary of them is presented in Table 3.

**Table 3:** Overall Euclidean distance / shape similarity score (%) and Hausdorff distance.

Groups	Number of masses	Euclidean distance / Shape similarity score (%)			Hausdorff distance		
		Convex-NMF	Sauwen et al.	SSSE	Convex-NMF	Sauwen et al.	SSSE
A and B	1	99.72 ± 24.99 / 79.07 ± 11.62	84.19 ± 14.46 / 85.72 ± 5.15	73.88 ± 17.67 / 88.75 ± 5.43	9.40 ± 1.35	8.22 ± 1.65	8.18 ± 1.49
C	1	122.23 ± 34.23 / 73.14 ± 14.52	102.41 ± 22.93 / 81.63 ± 7.57	76.76 ± 27.32 / 88.98 ± 8.18	10.22 ± 2.14	7.74 ± 1.80	6.61 ± 2.42

Distances between the produced colour-coded maps (Convex-NMF, Sauwen’s and SSSE) and the expert’s (mean and standard deviation reported per group). Columns 1 and 2 as in Table 2. Third to fifth columns include the Euclidean distance and the shape similarity score for the two approaches, respectively; while sixth to eighth show their corresponding Hausdorff distance. Shaded columns highlight the results obtained with the proposed methodology.

The results presented in Tables D and E in S1 File were tested for significance (comparing the three approaches against each other) using a Kruskal-Wallis test, to decide whether the population distributions are identical (null hypothesis) without assuming them to follow the normal distribution. They resulted in a p-value < 0.00001 (any p-value < 0.05 is deemed as significant).

## Discussion

This section mirrors the structure of the Results section, to facilitate the discussion.

### Brain tumour delineation

Starting with a discussion of the tumour delineation results for mice in Groups A and B, we can see that the high resemblance between the sources representing the same tissue type for each approach (Figs 4 to 6), is generally because the areas that they are representing are largely the same. However, the seemingly small differences displayed between them have an important impact in the resulting colour-coded maps. This is not surprising as we have seen this effect in the past where very small differences in the spectra led to the characterisation of the therapy response to temozolomide in preclinical glioblastoma in [26]. In this study, SSSE achieved a sharper delimitation of the tumour masses when comparing with Convex-NMF, probably due to the embedded information from the T2w image. Additionally, it is worth mentioning that the inclusion of the MRI information did not affect the biochemical interpretation of the sources, thanks to the way this information was embedded into the model. Moreover, SSSE outperformed its counterpart approach proposed by Sauwen et al. when used in equal conditions (i.e. same data), possibly because the initial information (labelling/segmentation) fed into SSSE is preserving better the spatial configuration of the information.

Overall, for mice in Group A, SSSE and Sauwen’s approach provided a more meaningful representation than Convex-NMF. This is especially relevant for mouse C179. Metabolically, regarding the spectral pattern represented, the yellow source obtained with Convex-NMF included metabolic features that would be expected both in tumour and non-tumour areas, such as high mobile lipids and high NAA, respectively. See relevant metabolites highlighted in Fig 1D and 1E).

In contrast, for mouse C179, SSSE yielded a source (coloured in yellow) that was very similar to the red (tumour) source (Fig 5). The main difference between them was a higher peak signal from lipids/lactate in the red source, which matched with the increased proliferation and necrosis, and the higher choline to creatine ratio (3.21:3.03 ppm). The choline signal had the same intensity of the lipid/lactate signal (1.28/1.33 ppm) in the yellow source, which is indicative of an active tumour, in agreement with the intermediate proliferation indices that were recorded in the yellow region. This goes in line to what we know about this case: this would be indeed expected, as the PIs calculated in zones inside this secondary mass are indicative of tumour tissue, with PI values above 20%. This difference in the spectral patterns are probably responsible for the production of the three coloured regions that closely matched the three histologically different regions. In the case of Sauwen’s approach, the obtained results are not too far from those obtained by SSSE, which reinforces the value of using any available knowledge (in this case MRI information) from the case/patient to guide the source extraction in a semi-automated/semi-supervised way.

Most importantly, in the case of Convex-NMF, when looking at the area in the colour-coded map that this yellow source is representing (Fig 5), it is considerably far from the secondary tumour mass and it is actually infiltrating non-tumour area in the bottom of the region of interest, which explains why this source exhibits some metabolic features of healthy tissue (see Fig 11 available at [25]). The latter is an important result as it shows that with both semi-supervised approaches, the system can be guided to find a representation of the knowledge that the researchers would need modelling, giving them a tool for testing different hypotheses–when left free in a completely unsupervised approach, the result will not necessarily relate to anything of interest as they could be separating regions (e.g. artefacts, ventricles, noise, etc.) that might not be relevant to the particular study.

**Fig 8:**
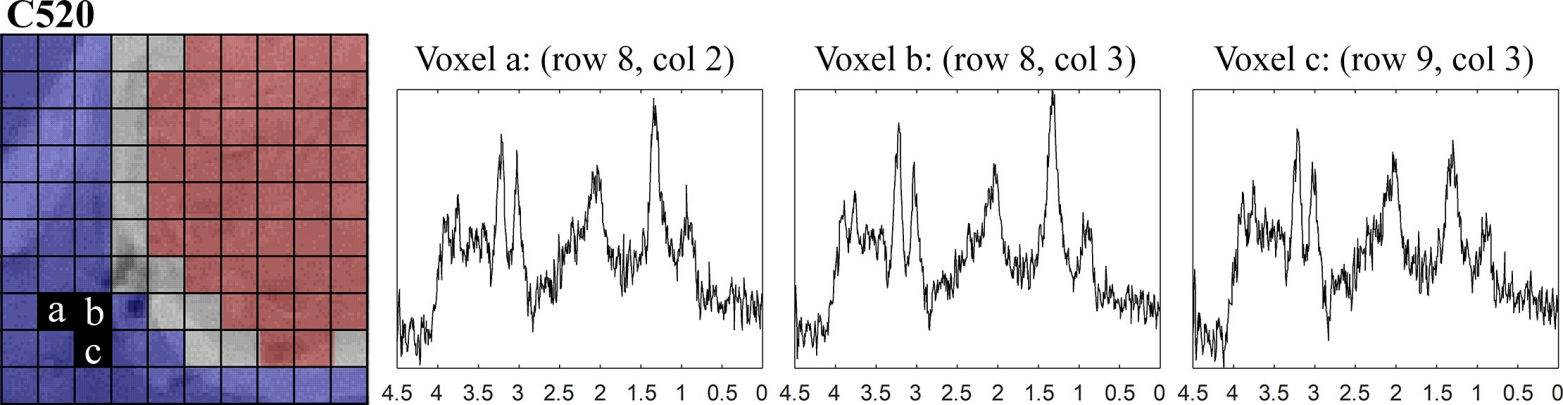
MR spectra of three selected voxels from the non-tumour area.

The results for Group B show that, in general, the spectral pattern exhibited a great variation among cases: for example, case C520, C529 and C583 show prominent peaks of lipids/lactate even in the non-tumour region, whereas case C255 and C288 did not. This is not totally unexpected as the first three cases had larger tumour volumes than the other ones, with most voxels within the MRSI grid being represented either by the tumour itself or by the peritumoral infiltrating zone. Fig 8 shows a selection of voxels from one of these mice, i.e. C520, in which a high peak of lipids/lactate at c.a. 1.3 ppm can be seen, especially prominent in voxels a and b which could be due to tumour tissue infiltration.

However, even for the cases in which the similarities between the sources are higher, again the resulting maps were quite different, as seen in the results reported for the Group A. Regardless of this apparent high similarity, the colour maps show a different representation of the tumour areas for each approach, which are visually more coincident with the initial manual segmentation, but with the benefit that they also include the researcher-dubious (grey/uncertain) areas.

In addition to all the aspects mentioned before, both semi-supervised approaches have shown the ability to learn more meaningful, better-quality sources, as a way to overcome the susceptibility to the presence of artefacts and the lower signal-to-noise spectra issues reported in [39], with SSSE providing more accurate results according to the gold standard.

The delineation of the tumour area in the maps for the Group C show that, as observed for Groups A and B, SSSE results in more coincidental areas with the abnormal regions. Additionally, they are in accordance with the RECIST criteria for each of these images. Indeed, for both mice, there was a much higher correspondence between the RECIST criteria, and the maps obtained when using SSSE.

### Evaluation of the tumour delineation against the gold standard

In most cases of Groups A and B, the sensitivity and specificity when using SSSE was better than the other two approaches (Table 2 and A in S1 File), and in some cases by a large difference. The exceptions of a higher sensitivity with Convex-NMF and/or Sauwen’s approach were seen in mice C179, C520 and C583, but these are at the cost of a much reduced specificity. If we look closer at mouse C520, for example, we can see that it showed some atypical features around the ventricle which the imaging expert marked as abnormal, although this does not necessarily mean that it corresponds to core tumour area, with PI > 30%. SSSE then benefitted from representing the tumour as a larger volume, but failed to do it accurately, as can be seen with a drop in specificity. In the case of specificity, SSSE also exhibited a better performance overall, only running aground with case C529 in which it also failed to identify the abnormal features around the ventricle (see Fig D in S1 File), as described by the preclinical bioimaging expert, possibly because they are not part of the core tumour area with PI > 30%.

When looking at the results of the analysis of the two abnormal masses from mouse C179 (see Fig 5 and Table 2 and B in S1 File), we can see that in cases such as this one, semi-supervised approaches can make a huge difference when creating a model that represents areas of interest (notice that the resulting areas in the colour map, first row of Fig 5, do not represent such areas), with overall sensitivity and specificity of SSSE (1.00 and 0.88, respectively) outperforming Sauwen’s approach (0.87 and 0.77, respectively) and Convex-NMF (0.70 and 0.68, respectively).

The sensitivity and specificity results for Group C are also consistent with the ones of Groups A and B. A detailed discussion for this group can be found in the Supporting Information file (Discussion of the tumour delineation sensitivity and specificity for Group C).

The Dice scores also show agreement with what was discussed previously. The semi-supervised approaches greatly outperform the unsupervised one, with SSSE exhibiting better performance. As recommended by Zijdenbos et al [41] in the literature of image validation, a good overlap occurs when DSC >0.70, which has been attained by the three approaches.

The Euclidean distance between the SSSE resulting maps and the gold standard were smaller in most of the cases than the distances between the maps obtained with the other two approaches and the gold standard (see Tables D and E in S1 File), which means that SSSE delineation of the tumour was more accurate than the other methods. There were only four exceptions to this (from all the 28 delineation maps produced in this study with the proposed methodology), two maps in group B, and another two in Group C. The ones in Group B were for mice C288 and C529, for which the Euclidean distances were slightly worse for SSSE, however the Hausdorff distance for both cases indicated otherwise. In addition, our proposed method did spot the anomaly in the ventricles of C529, which was also identified by the expert pathologist when analysing the samples *a posteriori*. The exceptions in Group C were case C819 at day 41 and case C821 at day 21, in which the differences were marginal, and again the Hausdorff distances contrarily show a better performance by SSSE. It is worth mentioning that the distance between two images will indicate how much they differ, meaning that the shorter the distance between them the higher their similarity.

Therefore, considering that a) most of the delineation maps produced by SSSE showed a notorious improvement over the unsupervised approach, and b) the maps produced by the counterpart, semi-supervised approach proposed by Sauwen et al. did not generally outperform SSSE, we can confidently say that overall, the distance of the maps produced by SSSE to the gold standard were considerably shorter, meaning that SSSE was better suited for the problem and data at hand.

Previous results for the Euclidean distances between the three approaches to the gold standard harmonise with those obtained for the shape similarity score, as these two evaluation methods are highly related, except that the latter provides a better understanding and interpretability of what these distances mean. Overall, for Groups A and B, the proposed methodology exhibited an improvement over the unsupervised approach in the shape similarity score of nearly 10%; and Group C showed an improvement of more than 15% (both with a much smaller standard deviation).

The results presented in Tables D and E in S1 File (referring to the Euclidean distance, similarity score, and Hausdorff distance between the three approaches to the gold standard) were tested for significance using a Kruskal-Wallis test, obtaining a p-value < 0.00001, which indicates that the difference between these approaches are statistically significant (as p < 0.05). Furthermore, statistically significant differences were found between the 28 delineation maps produced by the unsupervised approach and Sauwen’s approach in comparison to the 28 produced by SSSE.

One final note goes to the fact that the proposed methodology does not combine MRI and MRSI information by concatenation, nor it constrains the MRSI to fit the MRI segmentation, as opposed to several previous works, such as [3,20–22]. Instead, our proposed methodology embeds the information coming from the MRI (e.g. the manually selected areas) into the analysis of the MRSI by guiding the source extraction (which are MR spectra) in the direction of the areas of interest, according to the MR image. Our reason to avoid direct concatenation of the information from these MR approaches was to propose a model able to integrate them in a principled manner. Moreover, we use the full MR spectra in the usual range of interest [4.5–0 ppm], as opposed to quantifying only a selection of metabolites as in [3,20,21]. The added value of the latter is interpretability, providing not only the visualisation of the resulting map with the delineation of the tumour areas and healthy parenchyma, but also the spectral pattern associated to each of these segmented regions. This allows for extra validation and reassurance, and more importantly, better understanding of the results.

## Conclusions

Overall, the quality of the resulting colour-coded maps indicating the tumour delineation with the proposed methodology was considerably better than when using only Convex-NMF in a completely unsupervised approach, as in [25], or even an alternative semi-supervised approach proposed by Sauwen et al. [22]. This was carefully assessed in a quantitative way, showing that, in most cases, the sensitivity and specificity of delineating the tumour masses (which provide a measure of accuracy and confidence in these delineations) was far superior when using the proposed methodology, SSSE. These results were also consistent with the shape similarity scores and distances calculated for both sets of images (in both approaches) to the gold standard (images from the imaging expert), which can be considered statistically significant.

These results also come with additional advantages, namely: a) the proposed methodology is able to effectively deal with signal-to-noise issues, which is not the case of the unsupervised approach in [25]; b) it allows radiologists/clinicians to define the area of interest to them and, with that, guide the process of source extraction, which was not a possibility in [25]; c) while still creating an intra-subject model, as opposed to [26] where the model was trained using a set of subjects, therefore needing to deal with the tumour heterogeneity of GB, which is a well-recognised problem [42], and d) in addition to a segmented images, SSSE also produce meaningful, good-quality sources that represent each of the regions of interest, which adds an extra layer of interpretability to the results obtained, conferring not only validation of the results, but also better understanding and analysis of each individual case.

Therefore, given the quality of the obtained results, and the advantages identified in the use of the proposed methodology, we consider that the extra pre-processing steps related to the embedding of the MRI information into the MRSI data source extraction are worthwhile, as they have shown to improve the tumour delineation in the preclinical GB model. Once again, the multiparametric character of the proposed approach (fusing MRI and MRSI) has shown to provide better results compared with using a single approach (MRSI) [26].

## Supporting information

S1 File Supporting information: Embedding MRI information into MRSI data source extraction improves brain tumour delineation in animal models. This document includes additional information regarding the Magnetic Resonance studies, TMZ administration and preparation, how Sauwen et al. method was used, how the initial segmentation was performed, and further details on the evaluation and discussion of the results.
(DOCX)

## Abbreviations

FI: Fisher Information
GB: Glioblastoma
MDS: Multi-dimensional Scaling
MLP: Multi-Layer Perceptron
MR: Magnetic Resonance
MRI: Magnetic Resonance Imaging
MRS: MR spectroscopy
MRSI: Magnetic Resonance Spectroscopic Imaging
NMF: Non-negative Matrix Factorisation
PI: proliferation index
RECIST: response evaluation criteria in solid tumours
SSSE: semi-supervised source extraction
T2w: T2-weighted
