# Supplementary material for: Embedding MRI information into MRSI data source extraction improves brain tumour delineation in animal models
Source: PLoS One. 2019 Aug 15;14(8):e0220809. doi: 10.1371/journal.pone.0220809 (PMC6695141; doi:10.1371/journal.pone.0220809)
Supplement: S1 File — This document includes additional information regarding the Magnetic Resonance studies, TMZ administration and preparation, how Sauwen et al. method was used, how the initial segmentation was performed, and further details on the evaluation and discussion of the results. (DOCX) [file pone.0220809.s001.docx]

**Supporting Information: Embedding MRI information into MRSI data source extraction improves brain tumour delineation in animal models**

Sandra Ortega-Martorell; Ana Paula Candiota; Ryan Thomson; Patrick Riley; Margarida Julia-Sape; Ivan Olier.

# Magnetic Resonance studies, acquisition parameters

Tumours were induced by intracranial stereotactic injection of 10^5^ GL261 glioma cells in the caudate nucleus, as previously described in (1). Extensive details about the MRI and MRSI acquisition parameters are described in (1,2). Briefly, horizontal, high-resolution T2-weighted (T2w) MRI (TR/TEeff = 4200/36 ms) was acquired using a RARE (Rapid Acquisition with Relaxation Enhancement) sequence; field of view (FOV), 19.2 x 19.2 mm; number of slices, 10; number of averages (NA), 4. The T2w MRI resolution ranged from 75 x 75 to 150 x 150 μm/pixel and slice thickness ranged from 0.5 to 1mm, depending of the studied case.

MR spectroscopy provides useful and rich biochemical information about the metabolic state of tumours and can be performed at different echo times (TE), which are broadly divided into two groups, short and long TE. MRSI for the mice used in this study were acquired at short TE (12-14 ms), using a 2D CSI (Chemical Shift Imaging) sequence with PRESS localization, where: FOV, 17.6 x 17.6 mm; Volume of Interest (VOI), (5.5 x 5.5 x 1.0 mm) and with ST, 1mm; TR, 2500ms; SW, 4006.41Hz; NA, 512. Water suppression was performed with VAPOR, using a 300 Hz bandwidth (1,2). Short TE typically show more complex patterns, including signals from metabolites with short and long T2 relaxation times, such as lipids, glutamine/glutamate, and myoinositol, with an overall higher signal to noise ratio of the spectra.

# TMZ administration and preparation

TMZ was administered to mice with an intragastric probe (20 G, 38 mm, Popper & Sons, New York, USA) in three cycles of 5, 2 and 2 days interleaved with 3-day intervals. The periods of administration were days 11 to 15 post-implantation, 19-20 and 24-25 post-implantation at a dose of 60 mg/kg per day of treatment. For this purpose, the stock TMZ solution was diluted in the administration vehicle (DMSO 10% in saline) and the volume administered was 200 μl per animal (taking 20 g per animal as mean weight).

# Summary of adapted RECIST criteria

- Progressive disease: tumour volume increased more than 20% with respect to previous exploration
- Stable disease: tumour volume unchanged, decreased (no less than 30%) or increased (no more than 20%) with respect to previous exploration
- Partial response: tumour volume decreases more than 30% with respect to previous exploration

# Using the method proposed by Sauwen et al. in (3)

Even when our methodological approach differs from Sauwen et al. in (3), there are some similarities in relation to the aims and problem solved. However, we would like to emphasise that SSSE does not directly use the T2w MRI; instead, it uses the area manually drawn by the experts (who looked at the MRI) to inform the selection of voxels from those areas of interest. This is the reason why we say that in our study the information is embedded, but has not been directly concatenated.

Following advice from the research group where the method was developed, we downloaded from <https://www.esat.kuleuven.be/stadius/software.php> the “NMF Segmentation GUI”. We then used it as follows: As Sauwen’s method (3) heavily relies on a number of sophisticated steps prior to the NMF analysis, as part of the initialisation of its matrices (details can be found in the “NMF initialization” section of the original paper in (3)), we decided to include all pre-processing steps described in (3) that we could possibly apply to our data, in order to avoid an unfair comparison.

It is worth stressing that we applied Sauwen’s method to the same voxels that we used to create our initial MLP model. These voxels are used in Sauwen’s approach to fine-tune initial sources that will be used to initialise the NMF analysis. Therefore, in this sense, both methods were tested using the exact same data available, so that none of them is disadvantaged in this comparison.

We must also remark one relevant difference between the way that we apply Sauwen’s method and its original publication: in our work we are not using the 18 image features as they do. There are three main reasons for this:

1. As mentioned previously, our methodology uses the T2w image only to have an indication for the initial selection of voxels, but after that we do not concatenate any of the image features with the spectra.
2. In (3), several different available MRI modalities were used. This is obviously different from our approach in which only T2w images are used. Also, as we require less information to produce a competitive model, this can be advantageous in practice as it would require the acquisition of less MR modalities.
3. The 18 image features initially extracted are not indicated/described in (3), but this is not critical to us since we are proposing a different approach that does not require reproducing this part.

# Initial segmentation of the T2w images

In this section we detail how the initial segmentations were performed in this study and what kind of considerations were taken into account.

For the initial segmentation of the image, we manually selected the areas of interest only following the anatomical criteria (by visual inspection of the T2w image) in all three groups for the selection of the areas of interest. We chose to use in this study a starting point based on a manual selection of these areas of interest, since it allows us to a) be more specific in the kind of questions we wanted to address, e.g. when we wanted to consider the analysis of one or two different masses, such as the case of C179 that contains a second small “cap” of less proliferative cells situated top left of the main mass; and b) to be able to discard areas of ventricles more effectively, as sometimes the intensity level of both, the ventricles and the tumour masses, can be similar, and automatic tools have shown to struggle in their differentiation.

Figures A and B show exploratory information from mice C69 and C179 that led to the initial segmentation of these two cases. Specifically, regions of interest of both mice are shown on the left, indicating which areas were potentially different, as judged by visual inspection at the T2w image, to the eye of the researcher. Representative spectra from these areas are also shown in these figures (in the middle), together with the mean spectra and standard deviation of the same areas (on the right). This can provide the reader with an idea of how these spectra look like, but it is worth mentioning that this information was not taken into account by the researchers to produce the initial segmentations. The MR spectra in these groups are the information that will be ultimately used by our proposed machine learning methodology to produce a final colour-coded image representing the different areas of interest, such as tumour, non-tumour, etc., while embedding the morphological information from the MR image.

**Fig A**. Region of interest of mouse C69. Left: T2w image enlarged and overlaid with the MRSI matrix at short TE with spectra shown in white. Manual segmentation by the researcher led to the overlaid map where red represents tumour and blue non-tumour. Middle: Two selected voxels, 1 and 2, which are representatives of the two tissue types, are shown enlarged. Right: mean spectra of the two areas, red for tumour and blue for non-tumour, and their corresponding standard deviation in grey.

**Fig B**. Region of interest of mouse C179. Left: T2w image enlarged and overlaid with the MRSI matrix at short TE with spectra shown in white. Manual segmentation by the researcher led to the overlaid map where red and yellow represent two different tumour masses and blue non-tumour. Middle: Three selected voxels, 1, 2, and 3, which are representatives of the three tissue types, are shown enlarged. Right: mean spectra of the three areas: red and yellow for the tumour masses and blue for non-tumour, and their corresponding standard deviation in grey.

Figures C and D compile the initial segmentations for the mice in groups A and B, respectively.

**Fig C**. Initial segmentation of mice from Group A. Top row: MRI of the region of interest. Middle row: manual segmentation of the MRI by the researcher. Bottom row: images after lowering the resolution to match the one of the MRSI, i.e. from pixel level to voxel level.

**Fig D**. Initial segmentation of mice from Group B. Rows as in Fig C.

For cases in Group A (4), the pathologist had advised to consider tumour regions those with a PI > 30%, whereas non-tumour would correspond to those regions with a PI ≤ 5%, as mentioned previously in the Histopathology section. This was a safe threshold for all group cases ensuring consistent results. However, the second mass of mouse C179 (Fig B, yellow area) had a global PI < 30% (see Fig 9 in (4)), although small areas in that second mass showed a PI of 40%. The region between both masses had PIs = 21.5 - 24.5% (see Fig 11 in (4)). For these reasons, we decided to study two segmentations; one with two definite areas, i.e. Fig C – C179(a); and another one with three definite areas, i.e. Fig C – C179(b).

# Initial segmentation – discussion

We chose to start with a manual segmentation as we consider it to be more suitable for the kind of complexity we are bound to encounter in this type of data. For example, mouse C179 is very heterogeneous: it had two tumour masses (4), the first (biggest) with a high PI of 62.7%, and the second mass with a lower PI of 40%, with an intermediate area linking both with approximately 20 - 25% PI. The T2w image also shows parts of the ventricles with cerebrospinal fluid, which intersects with both masses. For this reason, we decided to omit in this study any segmentation approach based on pixel intensities, as we have seen that they can consider ventricles as if they were part of the tumour area, which is understandable due to their lighter appearance in the T2w images. Therefore, we considered that these approaches were not appropriate for this study as they are unable to account for the presence of this type of structures and decided to only use manual segmentations as the starting point of our proposed semi-supervised approach.

Using manual segmentations was considered a further advantage in this study because, more often than not, radiologists or clinicians have a very good idea of what are the different structures found in the T2w MRI, e.g. ventricles, an abnormal mass, etc. However, things get more complex when they need to identify the borders between them, e.g. in mouse C179, the border between the ventricles and the biggest tumour mass. Using this manual segmentation, they can provide the areas that they feel they can confidently identify, while leaving out any area of uncertainty. Additionally, this also facilitates performing different hypothesis testing, as the methodology can be applied to distinguish tumour vs. non-tumour, but also to distinguish between three areas (as in Fig 5), and even potentially more.

Importantly, even when using manual segmentations has been considered the best approach for this study, the proposed methodology can be applied using any form of initial segmentation, be it produced manually with the intervention of researchers, or automatically produced by software tools (provided that they produce an initial selection of areas that satisfy a problem of interest). Therefore, future studies may benefit from other types of automatic initial segmentations, e.g. those that take into account registered brain atlases (5), or a combination of both, assisted with manual segmentation.

# Details of the evaluation of the tumour mass delimitation

The sensitivity and specificity values corresponding to the ability of the three approaches to detect the tumour masses can be found in tables A (for Groups A and B), B (for more details on mouse C179 from Group A), and C (for Group C). These tables also include the Dice score coefficient calculated for these methods against the gold standard.

The sensitivity, specificity, and Dice score of the results presented in Fig 5, when considering the two abnormal masses from mouse C179, are shown in table B.

Table A. Sensitivity / specificity and Dice score of the correct delimitation of the tumour mass for Groups A and B. First column indicates the group; second indicates the subject ID (for comparison purposes with previous published studies); third contains the day post-inoculation (p.i.) of the mouse; fourth to sixth columns include the sensitivity / specificity results for the three approaches, respectively; while seventh to ninth show their corresponding Dice scores. Bottom row compiles the mean and standard deviation of columns 4^th^ - 9^th^. Shaded columns highlight the results obtained with the proposed methodology, SSSE.

| **Group** | **Mouse ID** | **Day p.i.** | **Sensitivity / Specificity** | | | **Dice score** | | |
| --- | --- | --- | --- | --- | --- | --- | --- | --- |
|  |  |  | **Convex NMF** | **Sauwen et al.** | **SSSE** | **Convex NMF** | **Sauwen et al.** | **SSSE** |
| A | C69 | 15 | 0.93 **/** 0.78 | 0.93 / 0.80 | 0.97 / 0.86 | 0.85 | 0.86 | 0.91 |
| A | C71 | 16 | 0.87 / 0.81 | 0.90 / 0.79 | 0.92 / 0.80 | 0.87 | 0.88 | 0.90 |
| A | C32 | 16 | 0.77 / 0.84 | 0.74 / 0.88 | 0.85 / 0.83 | 0.83 | 0.82 | 0.87 |
| A | C179 | 17 | 0.78 / 0.39 | 1.00 / 0.56 | 0.98 / 0.74 | 0.64 | 0.81 | 0.86 |
| A | C233 | 17 | 0.93 / 0.89 | 0.92 / 0.86 | 0.99 / 0.89 | 0.92 | 0.91 | 0.96 |
| A | C234 | 17 | 0.84 / 0.89 | 0.88 / 0.92 | 0.90 / 0.91 | 0.88 | 0.91 | 0.92 |
| B | C255 | 14 | 0.93 / 0.85 | 0.93 / 0.83 | 0.97 / 0.89 | 0.90 | 0.90 | 0.94 |
| B | C288 | 18 | 1.00 / 0.44 | 1.00 / 0.83 | 1.00 / 0.82 | 0.57 | 0.81 | 0.81 |
| B | C520 | 18 | 0.94 / 0.80 | 0.90 / 0.87 | 0.89 / 0.97 | 0.91 | 0.91 | 0.93 |
| B | C529 | 18 | 0.91 / 0.52 | 0.91 / 0.51 | 0.94 / 0.38 | 0.86 | 0.86 | 0.85 |
| B | C583 | 18 | 1.00 / 0.27 | 0.91 / 0.94 | 0.98 / 0.90 | 0.81 | 0.93 | 0.96 |
| **Overall (mean and standard deviation):** | | | **0.90 ± 0.08 / 0.68 ± 0.23** | **0.90 ± 0.08 / 0.76 ± 0.14** | **0.94 ± 0.05 /**  **0.82 ± 0.16** | **0.82 ± 0.11** | **0.84 ± 0.09** | **0.90 ± 0.05** |

Table B. Sensitivity / specificity and Dice score for mouse C179 (from Group A) when looking at the two tumour masses – see Fig 2, image C179(b). Columns of this table are similar to those in table A.

| **Group** | **Mouse ID** | **Tumour**  **mass** | **Sensitivity / Specificity** | | | **Dice score** | | |
| --- | --- | --- | --- | --- | --- | --- | --- | --- |
|  |  |  | **Convex NMF** | **Sauwen et al.** | **SSSE** | **Convex NMF** | **Sauwen et al.** | **SSSE** |
| A | C179 | Core mass | 0.40 / 0.71 | 0.74 / 0.85 | 1.00 / 0.84 | 0.40 | 0.72 | 0.85 |
| A | C179 | Secondary | 1.00 / 0.65 | 1.00 / 0.69 | 1.00 / 0.91 | 0.50 | 0.53 | 0.78 |
| **Overall (mean and standard deviation):** | | | **0.70 ± 0.42 / 0.68 ± 0.04** | **0.87 ± 0.18 / 0.77 ± 0.11** | **1.00 ± 0.00 /**  **0.88 ± 0.05** | **0.45 ± 0.05** | **0.63 ± 0.13** | **0.82 ± 0.04** |

Table C. Sensitivity / specificity and Dice score for Group C, longitudinal study. Columns as in table A.

| **Group** | **Mouse ID** | **Day p.i.** | **Sensitivity / Specificity** | | | **Dice score** | | |
| --- | --- | --- | --- | --- | --- | --- | --- | --- |
|  |  |  | **Convex NMF** | **Sauwen et al.** | **SSSE** | **Convex NMF** | **Sauwen et al.** | **SSSE** |
| C | C819 | 10 | 1.00 / 0.67 | 0.99 / 0.78 | 1.00 / 0.91 | 0.67 | 0.75 | 0.88 |
| C | C819 | 15 | 0.92 / 0.71 | 0.94 / 0.90 | 0.98 / 0.92 | 0.83 | 0.92 | 0.95 |
| C | C819 | 18 | 0.92 / 0.64 | 0.90 / 0.70 | 0.96 / 0.87 | 0.85 | 0.86 | 0.94 |
| C | C819 | 21 | 0.98 / 0.46 | 0.93 / 0.75 | 0.99 / 0.71 | 0.90 | 0.92 | 0.95 |
| C | C819 | 25 | 1.00 / 0.50 | 1.00 / 0.65 | 1.00 / 0.75 | 0.78 | 0.83 | 0.87 |
| C | C819 | 30 | 0.95 / 0.53 | 0.95 / 0.61 | 0.85 / 0.96 | 0.66 | 0.70 | 0.88 |
| C | C819 | 33 | 0.95 / 0.52 | 0.99 / 0.49 | 1.00 / 0.78 | 0.65 | 0.66 | 0.82 |
| C | C819 | 41 | 0.91 / 0.67 | 0.89 / 0.81 | 1.00 / 0.68 | 0.75 | 0.81 | 0.80 |
| C | C819 | 45 | 0.65 / 0.81 | 0.91 / 0.90 | 1.00 / 0.85 | 0.76 | 0.94 | 0.98 |
| C | C821 | 10 | 0.97 / 0.49 | 0.70 / 0.73 | 0.76 / 0.77 | 0.50 | 0.52 | 0.58 |
| C | C821 | 15 | 0.90 / 0.73 | 0.90 / 0.68 | 0.97 / 0.83 | 0.79 | 0.77 | 0.88 |
| C | C821 | 18 | 0.96 / 0.73 | 0.94 / 0.78 | 0.98 / 0.83 | 0.89 | 0.89 | 0.93 |
| C | C821 | 21 | 0.97 / 0.88 | 0.97 / 0.80 | 0.94 / 0.93 | 0.96 | 0.94 | 0.95 |
| C | C821 | 25 | 0.60 / 0.17 | 0.83 / 0.76 | 0.98 / 0.87 | 0.64 | 0.87 | 0.97 |
| C | C821 | 30 | 0.98 / 0.53 | 0.86 / 0.79 | 0.98 / 0.92 | 0.93 | 0.90 | 0.98 |
| C | C821 | 33 | 0.39 / 0.56 | 0.76 / 0.43 | 0.67 / 0.45 | 0.56 | 0.86 | 0.80 |
| **Overall (mean and standard deviation):** | | | **0.88 ± 0.17 / 0.60 ± 0.17** | **0.90 ± 0.08 / 0.72 ± 0.13** | **0.94 ± 0.10**  **/ 0.81 ± 0.13** | **0.76 ± 0.13** | **0.82 ± 0.12** | **0.89 ± 0.10** |

Next, detailed results of i) the Euclidean distance and shape similarity score are shown, and ii) the Hausdorff distance between the same set of images. The results for the cases in Groups A and B are shown in Table D; while the results for Group C can be seen in Table E.

Table D. Euclidean distance / shape similarity score (%) and Hausdorff distance between the produced colour-coded maps for the different approaches and the expert’s for Groups A and B. Columns 1 to 3 and bottom row as in table 2. Fourth to sixth columns include the Euclidean distance and the shape similarity score for the two approaches, respectively; while seventh to ninth show their corresponding Hausdorff distance. Shaded columns highlight the results obtained with the proposed methodology.

| **Group** | **Mouse ID** | **Day p.i.** | **Euclidean distance /**  **Shape similarity score (%)** | | | **Hausdorff distance** | | |
| --- | --- | --- | --- | --- | --- | --- | --- | --- |
|  |  |  | **Convex NMF** | **Sauwen et al.** | **SSSE** | **Convex NMF** | **Sauwen et al.** | **SSSE** |
| A | C69 | 15 | 83.5 / 84.8 | 80.3 / 86.0 | 64.2 / 91.0 | 9.80 | 6.25 | 6.08 |
| A | C71 | 16 | 90.5 / 84.3 | 86.2 / 85.7 | 81.6 / 87.2 | 8.60 | 8.60 | 10.05 |
| A | C32 | 16 | 104.5 / 79.4 | 105.5 / 79.1 | 91.6 / 84.2 | 10.49 | 9.64 | 9.90 |
| A | C179 | 17 | 141.3 / 57.6 | 104.2 / 77.0 | 83.5 / 85.2 | 10.86 | 10.96 | 9.17 |
| A | C233 | 17 | 69.7 / 91.1 | 74.1 / 90.0 | 53.5 / 94.8 | 10.72 | 7.48 | 10.15 |
| A | C234 | 17 | 90.5 / 85.7 | 78.5 / 89.3 | 75.3 / 90.1 | 7.87 | 7.55 | 6.93 |
| B | C255 | 14 | 74.0 / 88.8 | 74.8 / 88.7 | 58.1 / 93.1 | 7.42 | 7.21 | 7.21 |
| B | C288 | 18 | 135.4 / 59.4 | 75.0 / 87.5 | 76.1 / 87.2 | 11.83 | 6.08 | 5.83 |
| B | C520 | 18 | 76.9 / 88.7 | 76.5 / 88.8 | 64.6 / 92.0 | 8.31 | 8.31 | 8.30 |
| B | C529 | 18 | 104.9 / 79.1 | 106.1 / 78.6 | 111 / 76.6 | 8.78 | 10.77 | 8.72 |
| B | C583 | 18 | 125.7 / 70.9 | 64.9 / 92.2 | 53.2 / 94.8 | 8.72 | 7.55 | 7.62 |
| **Overall (mean and standard deviation):** | | | **99.72 ± 24.99 / 79.07 ± 11.62** | **84.19 ± 14.46 / 85.72 ± 5.15** | **73.88 ± 17.67 / 88.75 ± 5.43** | **9.40 ± 1.35** | **8.22 ± 1.65** | **8.18 ± 1.49** |

Table E. Euclidean distance / shape similarity score (%) and Hausdorff distance between the produced colour-coded maps with the different approaches and the expert’s for Group C, longitudinal study. Columns as in table D.

| **Group** | **Mouse ID** | **Day p.i.** | **Euclidean distance /**  **Shape similarity score (%)** | | | **Hausdorff distance** | | |
| --- | --- | --- | --- | --- | --- | --- | --- | --- |
|  |  |  | **Convex NMF** | **Sauwen et al.** | **SSSE** | **Convex NMF** | **Sauwen et al.** | **SSSE** |
| C | C819 | 10 | 119.4 / 75.3 | 98.0 / 83.4 | 62.5 / 93.2 | 7.94 | 6.56 | 6.16 |
| C | C819 | 15 | 103.4 / 81.1 | 68.7 / 91.2 | 52.4 / 95.1 | 9.33 | 5.92 | 5.39 |
| C | C819 | 18 | 107.5 / 80.8 | 103.7 / 82.2 | 66.2 / 92.7 | 12.08 | 6.63 | 4.47 |
| C | C819 | 21 | 101.4 / 84.5 | 89.9 / 87.8 | 74.5 / 91.6 | 9.11 | 7.68 | 4.47 |
| C | C819 | 25 | 131.5 / 73.3 | 110.6 / 81.1 | 93.4 / 86.5 | 12.17 | 6.08 | 6.00 |
| C | C819 | 30 | 141.7 / 67.2 | 130.3 / 72.3 | 68.0 / 92.5 | 9.54 | 6.86 | 4.90 |
| C | C819 | 33 | 148.1 / 66.1 | 149.5 / 65.5 | 96.6 / 85.6 | 9.75 | 9.54 | 6.40 |
| C | C819 | 41 | 119.0 / 76.6 | 99.4 / 83.7 | 108 / 80.7 | 8.66 | 6.56 | 6.00 |
| C | C819 | 45 | 135.9 / 69.3 | 75.1 / 90.6 | 47.5 / 96.2 | 11.86 | 11.83 | 5.83 |
| C | C821 | 10 | 151.5 / 59.0 | 124.9 / 72.1 | 113.7 / 76.9 | 10.86 | 7.55 | 7.62 |
| C | C821 | 15 | 110.1 / 79.9 | 118.0 / 76.9 | 81.1 / 89.1 | 9.59 | 8.83 | 7.14 |
| C | C821 | 18 | 90.6 / 85.9 | 86.6 / 87.1 | 70.8 / 91.4 | 9.59 | 6.63 | 7.14 |
| C | C821 | 21 | 56.9 / 94.0 | 67.7 / 91.5 | 58.0 / 93.7 | 9.95 | 7.42 | 8.12 |
| C | C821 | 25 | 171.1 / 49.2 | 104.6 / 81.0 | 51.0 / 95.5 | 12.85 | 8.00 | 7.14 |
| C | C821 | 30 | 77.6 / 88.6 | 89.7 / 84.7 | 43.3 / 96.4 | 5.39 | 6.33 | 4.12 |
| C | C821 | 33 | 189.9 / 39.4 | 122.0 / 75.0 | 141.2 / 66.5 | 14.87 | 11.36 | 14.87 |
| **Overall (mean and standard deviation):** | | | **122.23 ± 34.23 / 73.14 ± 14.52** | **102.41 ± 22.93 / 81.63 ± 7.57** | **76.76 ± 27.32 / 88.98 ± 8.18** | **10.22 ± 2.14** | **7.74 ± 1.80** | **6.61 ± 2.42** |

# Discussion of the tumour delineation sensitivity and specificity for Group C

The sensitivity and specificity results for Group C (Tables 2 and C) are also consistent with the ones of Groups A and B. Generally, SSSE outperforms the other two methods. There are only a few cases in which the sensitivity decreases for the proposed methodology, because the representation of the tumour mass in the other maps was covering much wider areas, which included the tumour masses but also much of their surrounding tissue (see Fig 7), resulting in poorer specificity. As per the specificity, only in few occasions SSSE was not the best choice, for example case C821 at day 33, just prior to death/ethical euthanisation of this mouse. A closer look at this case would show that, at this point, all the region of interest was essentially covered by the tumour according to the imaging expert (see Fig 2), with RECIST criteria indicating a stage of progressive disease. Therefore, we consider that most probably all methodological approaches were learning and representing the intra-tumoural heterogeneity of this case.

In the case C819 at day 41, the results were calculated assuming solid tumour to be the area delineated in red (please refer to Fig 2), and not only visually the resulting map seems better (Fig 7), but also the sensitivity increases with SSSE, while maintaining the same level of specificity. If the blue area highlighted by the pathologist (Fig 2) for this particular case and day was considered part of the tumour, then the sensitivity would be 0.92 and 1.00 for Convex-NMF and SSSE approaches, respectively, which means that they remain almost the same; however the specificity would increase to 0.77 and 0.79 for Convex-NMF and SSSE, respectively, which represents an increase of approximately 10% for both methods.

# References

1. Simões R V., Delgado-Goñi T, Lope-Piedrafita S, Arús C. 1H-MRSI pattern perturbation in a mouse glioma: the effects of acute hyperglycemia and moderate hypothermia. NMR Biomed. 2010;23(1):23–33.

2. Delgado-Goñi T, Ortega-Martorell S, Ciezka M, Olier I, Candiota AP, Julià-Sapé M, et al. MRSI-based molecular imaging of therapy response to temozolomide in preclinical glioblastoma using source analysis. NMR Biomed. 2016;29(6):732–43.

3. Sauwen N, Acou M, Sima DM, Veraart J, Maes F, Himmelreich U, et al. Semi-automated brain tumor segmentation on multi-parametric MRI using regularized non-negative matrix factorization. BMC Med Imaging. 2017;17(1):29.

4. Ortega-Martorell S, Lisboa PJG, Simões R V, Pumarola M, Julià-Sapé M, Arús C. Convex Non-Negative Matrix Factorization for brain tumor delimitation from MRSI data. PLoS One. 2012;7(10):e47824.

5. Luts J, Laudadio T, Idema AJ, Simonetti AW, Heerschap A, Vandermeulen D, et al. Nosologic imaging of the brain: segmentation and classification using MRI and MRSI. NMR Biomed. 2009;22(4):374–90.
